# Reaffirming the Consistency of the Original Stock of NBS 19 Limestone and Its Byproduct With Finer Grain Size for *δ* ^13^C and *δ* ^18^O Calibrations—Both Distributed Internationally

**DOI:** 10.1002/rcm.70131

**Published:** 2026-07-01

**Authors:** Haiping Qi, Tyler B. Coplen, Lauren T. Reid, Stanley J. Mroczkowski

**Affiliations:** ^1^ U.S. Geological Survey Reston Virginia USA

**Keywords:** carbon and oxygen isotopes, Toilet Seat (TS) limestone, traceability, TS Marble

## Abstract

**Rationale:**

Approximately 3–4 kg of NBS 19 limestone, prepared in 1982 by the U.S. Geological Survey (USGS), serves as a primary reference for the carbon isotope scales VPDB and VPDB‐LSVEC and the oxygen isotope scale VPDB. Its consensus values are +1.95‰ for *δ* ^13^C and −2.2‰ for *δ* ^18^O measurements. After more than three decades of distribution, the number of units labeled “NBS 19” shrunk substantially, and the remaining material was quarantined, with a small fraction retained at the USGS and a larger fraction entrusted to the International Atomic Energy Agency (IAEA) for secure storage. Given the critical role of NBS 19, verifying the integrity of its *δ* ^13^C and *δ* ^18^O values across these storage locations is essential.

**Method:**

Samples from IAEA and USGS stock materials were selected to ensure traceability of carbon and oxygen isotopes to NBS 19 distribution units from various storage locations. To achieve this, distribution units labeled as NBS 19 from both the IAEA and the National Institute of Standards and Technology (NIST) were included. During evaluation, two distinct grain sizes were identified within these units. Grain size was assessed using sieves on five selected samples. The *δ* ^13^C and *δ* ^18^O values of finer and coarser fractions (both labeled NBS 19) were measured at USGS using continuous‐flow isotope‐ratio mass spectrometry on 100‐ and 200‐μg samples.

**Results:**

Expanded 95% measurement uncertainties (*k* = 2) for *δ* ^13^C and *δ* ^18^O determinations are 0.027‰ and 0.038‰ or better, respectively, for quarantined NBS 19 stocks and finer byproduct calcite, which we term “NBS‐19 byproduct,” regardless of storage location. Between the 1990s and circa 2011, NBS‐19 byproduct labeled “NBS 19,” distributed by NIST and the IAEA, shows these findings.

**Conclusions:**

NBS 19 primary isotopic reference material should be used for calibration of new isotopic reference materials rather than NBS‐19 byproduct secondary isotopic reference material.

## 1 Introduction

Stable isotope‐delta measurements of carbon and oxygen (expressed commonly as *δ* ^13^C and *δ* ^18^O) are vital tools employed across a diverse range of scientific fields, including anthropology, atmospheric sciences, biology, chemistry, environmental sciences, food and drug authentication, forensic science, geochemistry, geology, oceanography, and paleoclimatology [1]. These measurements offer critical insights into processes that encompass subjects from ancient climate patterns to contemporary environmental analyses. Isotope‐delta measurements are differential measurements that require a primary isotopic reference material (RM) having a permanently fixed value [2]. The first widely used RM for *δ* ^13^C and *δ* ^18^O measurements was belemnite from the Peedee Formation of South Carolina (PDB) collected by Heinz Lowenstam and Harold Urey [3]. The consensus *δ* ^13^C and *δ* ^18^O values of PDB were both zero.

**FIGURE 1:**
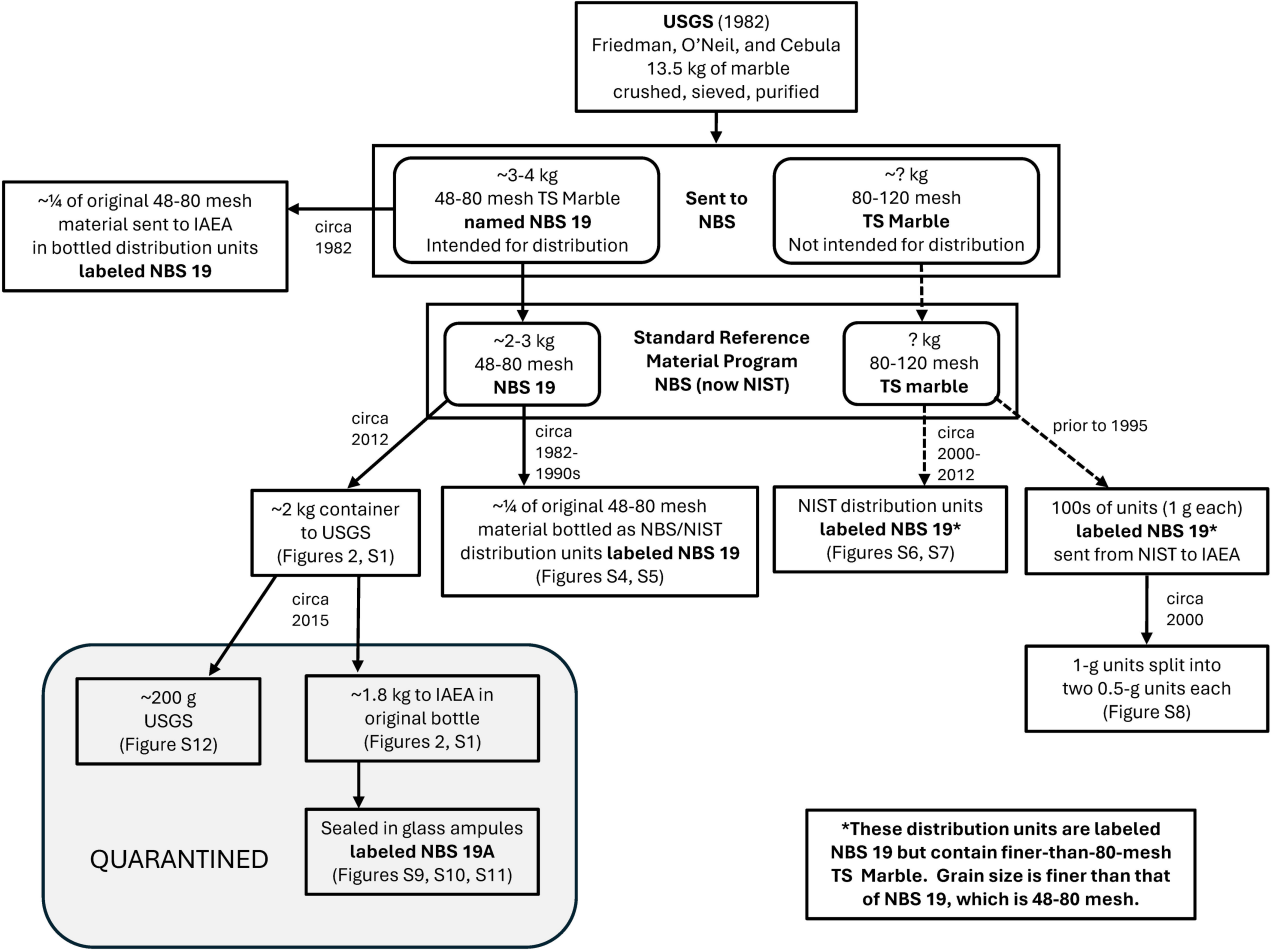
This flowchart represents the material flow of NBS 19 limestone [8] and its finer byproduct based on current understanding and documentation. This isotopic reference material was prepared by the U.S. Geological Survey [7] and sent to the National Bureau of Standards (NBS; now the National Institute of Standards and Technology [NIST]) and to the International Atomic Energy Agency (IAEA). Figures S1 through S12 are found in the Supporting Information [9]. Dotted lines indicate assumed transfers without documentation.

**FIGURE 2:**
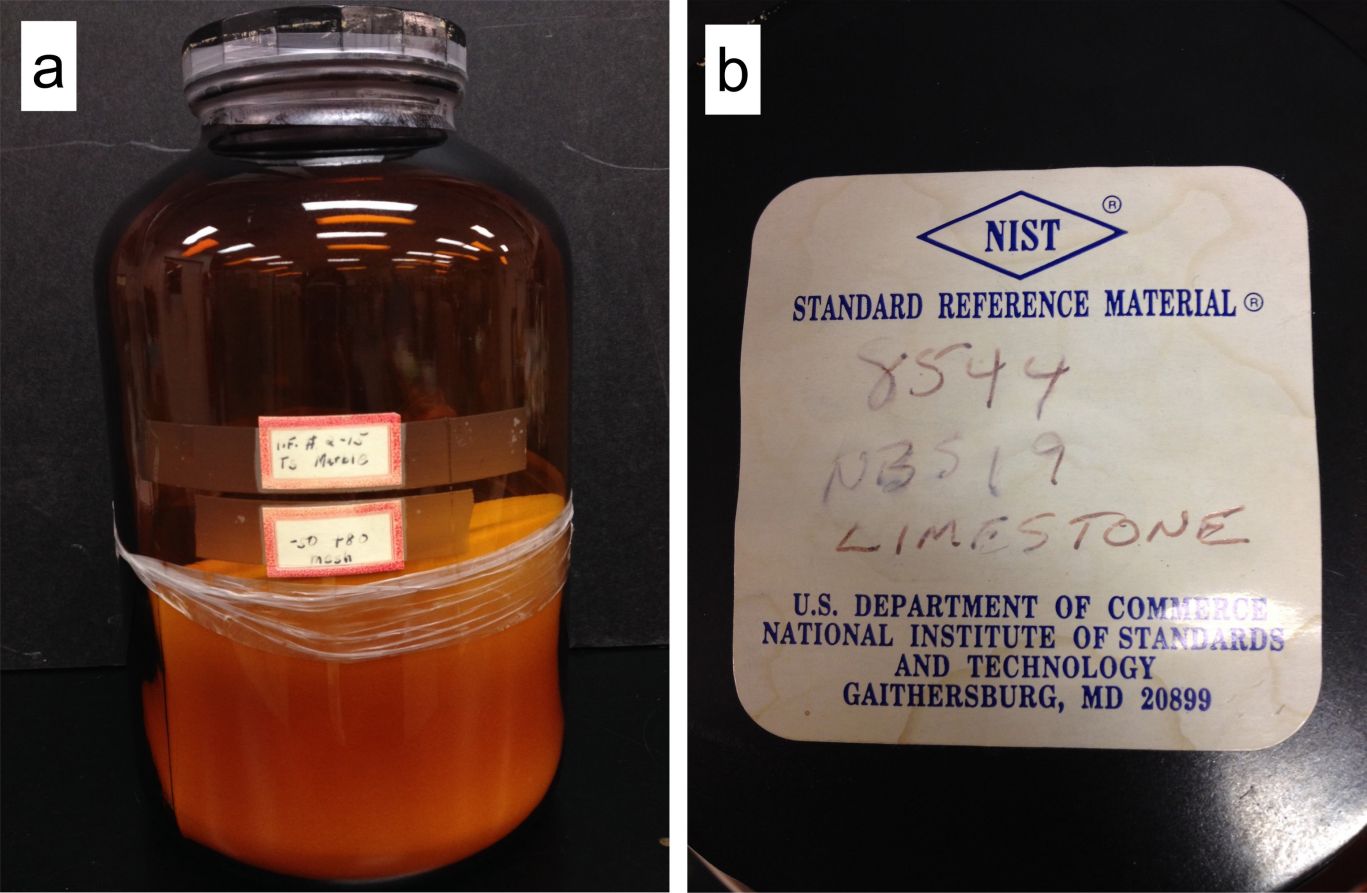
Original container of NBS 19 limestone provided to the National Bureau of Standards (now the National Institute of Standards and Technology [NIST]) by the U.S. Geological Survey (USGS). (a) Photo of USGS container (brown bottle) of NBS 19 returned to the USGS from NIST in 2012, showing the original labels of Friedman et al. [7]. Photo credit: Haiping Qi. (b) Photo of label on the black cap identifying the contents as NIST RM 8544, NBS 19 limestone. Photo credit: Haiping Qi.

At a consultants' group meeting on stable isotope standards and intercalibration in hydrology and in geochemistry at the International Atomic Energy Agency (IAEA) in September 1976, Irving Friedman and James O'Neil of the U.S. Geological Survey (USGS) agreed to prepare a high‐purity calcite RM having stable carbon and oxygen isotopic compositions as close as possible to PDB, whose supply was exhausted [4, 5]. A single 13.5‐kg polished slab of marble (known colloquially as “Toilet Seat limestone” or “TS limestone” [6]) that had served as an isotopic RM for many laboratories was crushed, sieved, and purified [7]. The crushing, sieving, and purification of TS limestone resulted in 3–4 kg of high‐purity calcite having a grain size of 48‐to‐80 mesh (297 to 177 μm) [7], and this material was labeled “TS Marble 50 [to] 80 mesh” (Figures 1, 2a, S1, and S2) [9]. An unspecified quantity of byproduct material having a finer grain size (80 to approximately 120 mesh) was produced and may have been labeled “TS Marble 80 – 120 mesh” based on the information below. Crushing the slab can invariably generate a substantial quantity of high‐quality, finer‐grain marble than that sought. For example, in the preparation of about 19 kg of USGS19 calcite having a grain size of 48‐to‐80 mesh (297 to 177 μm) [10, 11], about 13 kg of USGS20 byproduct high‐purity calcite having a grain size of 80‐to‐200 mesh (177 to 74 μm) was produced. The material flow of NBS 19 limestone and its finer byproduct presented in Figure 1 is based on current understanding and documentation. The fine‐grained byproduct of NBS 19 that is akin to USGS20 does not have a name, and it can be termed “finer‐than‐80‐mesh TS Marble” or “NBS‐19 byproduct” as shown in Table 1.

**TABLE 1:** Carbon and oxygen isotopic abundances of samples analyzed in this study.

Lab ID	Identification by Friedman et al. [7]	Name	Sample description	Figure	*δ* ^13^C_VPDB_		*δ* ^18^O_VPDB_		*n*
					Value (‰)	*U*_95_ (‰)	Value (‰)	*U*_95_ (‰)	
C‐14316	NBS 19	TS Marble 48‐to‐80 mesh	IAEA Stock #1, quarantined NBS 19	S10	+1.951	0.023	−2.235	0.035	17
C‐14317	NBS 19	TS Marble 48‐to‐80 mesh	IAEA Stock #2, quarantined NBS 19	S11	+1.963	0.020	−2.208	0.038	18
C‐14318	None (NA)	Finer‐than‐80‐mesh TS Marble (NBS‐19 byproduct)	IAEA distribution unit	S8	+1.936	0.025	−2.190	0.032	18
C‐14319	NBS 19	TS Marble 48‐to‐80 mesh	USGS stock, quarantined NBS 19	S12	+1.958	0.023	−2.215	0.033	18
C‐14320	None (NA)	Finer‐than‐80‐mesh TS Marble (NBS‐19 byproduct)	NIST distribution unit #1	S6	+1.946	0.022	−2.197	0.038	18
C‐14321	NBS 19	TS Marble 48‐to‐80 mesh	NIST distribution unit #2	S4	+1.957	0.018	−2.182	0.028	28
C‐14322	NBS 19	TS Marble 48‐to‐80 mesh	NIST distribution unit from Stroud	S5	+1.937	0.027	−2.187	0.032	18

*Note:* Samples analyzed by continuous‐flow isotope‐ratio mass spectrometry measurement of about 100 and 200 μg of calcite using a Thermo Fisher GasBench II.

Abbreviations: NA, not applicable; Stroud, Stroud Water Research Center, Avondale, Pennsylvania; TS, Toilet Seat; *U*_95_, the expanded 95% combined uncertainty (*k* = 2), reflecting laboratory analytical variability and method/instrument uncertainty.

Based on the information provided in this study, both size fractions were sent to the National Bureau of Standards (NBS; now the National Institute of Standards and Technology [NIST]) in Gaithersburg, Maryland. Once received, the fraction having the grain size of 48‐to‐80 mesh was assigned the name NBS 19 limestone (Figures 2b and S3). Half of it was retained in the container provided by the USGS (Figures 2a,b, S1, and S2), and the remainder was bottled and shared with the IAEA [7]. This RM was distributed by NIST as RM 8544 (Figures S3 and S5) [8] with 0.4 g of calcite in each unit (Samples C‐14321 [Figure S4] and C‐14322 [Figure S5] in Tables 1 and S1) and by the IAEA with 0.5 g of calcite in each unit. Friedman et al. [7] prepared NBS 19 from marble, which can be some of the purest limestones [12], removing albite, quartz, dolomite, pyrite, and other minerals using heavy liquid separation with bromoform, followed by Frantz electromagnetic separation to produce high‐purity calcite. Figure 2a indicates that NBS 19 is 50‐to‐80 mesh; however, Friedman et al. [7] indicate that this RM is 48‐to‐80 mesh. The difference in grain size is likely due to the use of the Tyler standard mesh scale for 297‐μm grains for the publication (48 mesh) and the US standard mesh scale for the laboratory work (No. 50) [13]. Both scales are identical for 177‐μm grains (80 mesh and No. 80).

In 1987, with the exhaustion of PDB, NBS 19 was assigned permanent *δ* ^13^C and *δ* ^18^O values of +1.95‰ and −2.2‰ by international agreement for isotope‐delta scales named Vienna PDB (VPDB), for *δ* ^13^C_VPDB_ measurements of all carbon‐bearing materials and for *δ* ^18^O_VPDB_ measurements of carbonates [14, 15]. In 2006, a second primary isotopic RM material, LSVEC lithium carbonate, was adopted to create the *δ* ^13^C_VPDB‐LSVEC_ scale [16, 17] enabling two‐point isotope‐scale normalization [18]. Therefore, NBS 19 serves as an anchor for two *δ* ^13^C scales and retains its value of +1.95‰ for each [10, 11].

When the supply of distribution units of NBS 19 was getting low, new units were filled and distributed by NIST with byproduct from the finer‐than‐80‐mesh container that may have been labeled “TS Marble” (Sample C‐14320 in Tables 1 and S1; Figures S6 and S7) instead of calcite from the 48‐to‐80‐mesh container labeled TS Marble (Figures 2a and S1). Hundreds of these units containing finer‐than‐80‐mesh TS Marble were sent by NIST to the IAEA (Figures 1 and S8). They were distributed by the IAEA between approximately 2000 and 2011 with the label “NBS 19” (Figure S8) [9].

After widespread use, the number of distribution units dwindled, and NBS 19 was “quarantined” in 2011 [2] (Figure S8), which means that NIST and the IAEA terminated distribution of NBS 19 and retained the remaining material for future calibration needs. The bottle of remaining NBS 19 (Figures 2a,b and S1–S3) was returned to USGS by NIST in 2012. In December 2014, the quarantined status of NBS 19 was discussed at an IAEA meeting on isotopic RMs in Vienna, Austria. It was suggested to one of us (TBC) at this meeting that the bottle of NBS 19 (Figure 2a) might be more protected for long‐term storage at the IAEA than at the USGS. Therefore, in 2015, a major fraction of the quarantined NBS 19 was sent by the USGS to the IAEA for secure quarantined storage. When the IAEA received the NBS 19, it was recognized that its grain size was larger than that of the finer‐than‐80‐mesh TS Marble they had been distributing since approximately 2000 (Figure S8), and the IAEA assigned the new name “NBS 19A” (Figure S10) to this original NBS 19 having a grain size of 48‐to‐80 mesh. The IAEA removed all of the quarantined NBS 19 from its bottle (Figure 2a) and sealed it in 1‐ and 25‐mL glass ampules (Figure S9). Although approximately 2 kg of this high‐purity RM remains in quarantined storage at the IAEA and the USGS, there may be differences in *δ* ^13^C and *δ* ^18^O values of NBS 19 from the different grain sizes and the different storage locations. The quarantined status of this RM signifies a cautious approach to safeguarding the integrity of this primary isotopic RM, whereas factors like storage conditions and grain size are considered as they may affect performance.

The aim of this project is to determine if the existing stocks and distribution units at the IAEA and the USGS are indistinguishable based on *δ* ^13^C and *δ* ^18^O measurements. Seven samples accomplished this task satisfactorily (Tables 1 and S1). Collecting NBS 19 from isotope laboratories around the world and ensuring that their isotopic compositions are identical within analytical uncertainty is outside the scope of this study.

## 2 Method

### 2.1 Samples

A total of seven calcite samples (Tables 1 and S1) from IAEA quarantined stocks (Samples C‐14316 [Figure S10] and C‐14317 [Figure S11]), an IAEA distribution unit (Sample C‐14318 [Figure S8]), USGS quarantined stock (Sample C‐14319 [Figure S12]), and NIST distribution units (Samples C‐14320 [Figure S6], C‐14321 [Figure S4], and C‐14322 [Figure S5]) were analyzed. These include NBS 19 and NBS‐19 byproduct from finer‐than‐80 mesh TS Marble. Two NIST distribution units (Samples C‐14320 and C‐14321) were selected from several distribution units stored at the USGS in Reston, Virginia, since before 1995. One sample obtained by the USGS from the Stroud Water Research Center, Avondale, Pennsylvania, in 2023, appears to be an original NIST distribution unit of NBS 19 limestone (Sample C‐14322 [Figure S5]). Each sample is identified in Tables 1 and S1 according to the definition provided by Friedman et al. [7] as either NBS 19 when it was 48‐to‐80 mesh or “None” when its grain size was finer‐than‐80 mesh.

### 2.2 Grain Size Evaluation

Clean 3‐in. (76‐mm) diameter sieves having meshes of 60 (250 μm), 80 (177 μm), 100 (149 μm), and 115 (125 μm; US sieve size No. 120) [13] were used to assess the grain size of calcite samples, following the strategy used by Friedman et al. [7].

### 2.3 Isotope Analysis

Calcite samples having masses of 100 or 200 μg were placed in pre‐cleaned 12‐mL round‐bottom borosilicate Labco vials (Part Number 9RK8W). The vials were carefully tilted to a horizontal position, and 4 drops (about 20 μL) of 100% phosphoric acid by weight (specific gravity = 1.902) were manually added to the inner shoulder of each vial. The vials were then capped with double‐wadded caps (Labco VC329) and purged with ultra‐high‐purity (UHP) helium for 10 min. After helium purging, the vials were tilted back to a vertical position, allowing the acid to flow down to the bottom and react with the calcite samples. The samples were allowed to react with the acid at homogeneous room temperature (at 22°C ± 0.5°C) for 24 h. The CO_2_ evolved from the reaction was analyzed for *δ* ^13^C and *δ* ^18^O values using a Thermo GasBench connected to a Thermo Delta Plus XP continuous‐flow isotope‐ratio mass spectrometer (referred to as the GasBench II‐CF‐IRMS system). The reported *δ* ^13^C and *δ* ^18^O values were normalized [18] to VPDB using the mean values of all seven NBS 19 samples analyzed in the same run. The oxygen isotopic phosphoric acid fractionation factors for calcite cancel out because NBS 19 and finer‐than‐80‐mesh TS Marble are the same minerals and were analyzed using identical treatment methods at the same temperature [19].

The carbon and oxygen isotopic homogeneity was assessed through four runs, each with four to six aliquots from each of the seven samples. Among these runs, three runs contained samples having masses of 200 μg, whereas one run contained samples having masses of 100 μg. A fifth run involved multiple analyses of samples weighing 200 μg from the same NBS 19 vial (identified as C‐12928 in Table S1) that has been used in the USGS Reston Stable Isotope Laboratory (RSIL) to document analytical uncertainty. An expanded (95%) combined uncertainty (*k* = 2) is based on the standard deviation of the analyses (three repetitions of CO_2_ analyses).

## 3 Results and Discussion

### 3.1 Grain‐Size Evaluation

The grain sizes of the calcite samples investigated in this study are presented in Table S1. The mesh of the samples can be categorized as either 48‐to‐80 (297 to 177 μm) or 80‐to‐120 (177 to 125 μm). Table S2 categorizes the masses and fractions of three samples of 48‐to‐80‐mesh calcite samples (C‐14317, C‐14319, and C‐14322) by (1) mass of sample with grain size finer than 177 μm, (2) mass of sample with grain size between 177 and 250 μm, (3) mass of sample with grain size greater than 250 μm, (4) mass of sample with grain size greater than 177 μm, (5) fraction with grain size less than 177 μm, (6) fraction with grain size between 177 and 250 μm, (7) fraction with grain size greater than 250 μm, and (8) fraction with grain size greater than 177 μm. Most of the calcite falls within the range of 48‐to‐80 mesh (Table S2). Only about 6% of the calcite is finer‐than‐80 mesh. Table S3 categorizes the masses and fractions of two samples (Samples C‐14318 and C‐14320) of 80‐to‐120‐mesh calcite categorized by (1) mass of sample with grain size between 125 and 149 μm, (2) mass of sample with grain size between 149 and 177 μm, (3) mass of sample with grain size greater than 177 μm, (4) mass of sample with grain size greater than 149 μm, (5) fraction of sample with grain size between 125 and 149 μm, (6) fraction of sample with grain size between 149 and 177 μm, (7) fraction of sample with grain size greater than 177 μm, and (8) fraction of sample with grain size greater than 149 μm. The majority (about 97%) of the calcite falls within the range of 80‐to‐120 mesh; therefore, these calcite samples are categorized as finer‐than‐80‐mesh TS Marble samples. We categorized two more samples, Samples C‐14316 (“IAEA Stock #1, quarantined NBS 19” [Figure S10]) and C‐14321 (“NIST distribution unit #2” [Figure S4]), in Table S1 as 48‐to‐80 mesh (297 to 177 μm) by counting the number of grains in a 200‐μg sample of calcite and comparing the average mass per grain with that of other calcite samples (Table S4). The average mass per grain of 48‐to‐80‐mesh NBS 19 limestone is about 18 μg, and that of 80‐to‐120‐mesh TS Marble is about 8 μg.

All calcite falls into one of two grain sizes (Tables 1 and S2–S5), namely, 48‐to‐80 mesh (297 to 177 μm) or finer‐than‐80 mesh (≤ 177 μm). Both size fractions have been distributed by NIST and the IAEA (Table 1). In Table 1, one of the NIST distribution units (C‐14320 [Figure S6]) and an IAEA distribution unit (Sample C‐14318 [Figure S8]) contain finer‐than‐80‐mesh TS Marble. The remaining samples in Table 1 are 48‐to‐80‐mesh NBS 19 as described by Friedman et al. [7]. These included all samples identified as quarantined NBS 19 (C‐14316, C‐14317, and C‐14319), NIST distribution unit #2 (C‐14321), and the NIST distribution unit from the Stroud Water Research Center (C‐14322).

Because the supply of IAEA distribution units of NBS 19 was getting low, in 2000, the IAEA split hundreds of 1‐g distribution units of NBS 19 that it had received in the 1990s from NIST (Figure S8). These 1‐g distribution units were received with NIST labels and contained finer‐than‐80‐mesh TS Marble, and IAEA divided each into two 0.5‐g distribution units with IAEA labels (Figure S8) [9]. Therefore, the source of the finer‐than‐80‐mesh TS Marble is NIST. NIST also distributed finer‐than‐80‐mesh TS Marble in units labeled “NBS No 19 TS LIMESTONE”; for example, see Figure S6.

There is no evidence to suggest that any of the 48‐to‐80‐mesh NBS 19 was ground to produce the finer‐than‐80‐mesh TS Marble. The only source for finer‐than‐80‐mesh TS Marble that the authors can envision is the USGS itself. During the production of a calcite RM, crushing a marble slab invariably generates a substantial quantity of finer‐grain marble than that sought. For example, in the preparation of about 19 kg of USGS19 calcite having a grain size of 48‐to‐80 mesh (297 to 177 μm; [10, 11]), about 13 kg of USGS20 byproduct calcite having a grain size of 80‐to‐200 mesh (177 to 74 μm) was produced. During the production of 48‐to‐80‐mesh TS limestone (NBS 19), the authors believe that the finer‐than‐80‐mesh byproduct was saved in a separate container and that containers of both grain sizes were sent by the USGS to NBS (now NIST). If the container of finer‐than‐80‐mesh TS Marble was similar to that of 48‐to‐80‐mesh TS Marble (Figure 2a,b), removing calcite from the wrong container to prepare new units can explain how NIST sent hundreds of 1‐g NBS 19 distribution units to IAEA in the 1990s containing finer‐than‐80‐mesh TS Marble.

When the USGS provided its brown bottle of quarantined NBS 19 (Figures 2a,b and S1–S3) to the IAEA for secure quarantined storage in 2015, it was recognized by the IAEA that the grain size was larger than that of the material that they had been distributing since about 2000 [9]. Based on this observation, the IAEA assigned the new identifier “NBS 19A” (Figure S10) to the calcite in this bottle. This can explain the inclusion of “NBS19A” or “NBS 19A” in identifiers of IAEA samples (Figures S10 and S11).

### 3.2 Results of *δ* ^13^C and *δ* ^18^O Measurements

**FIGURE 3:**
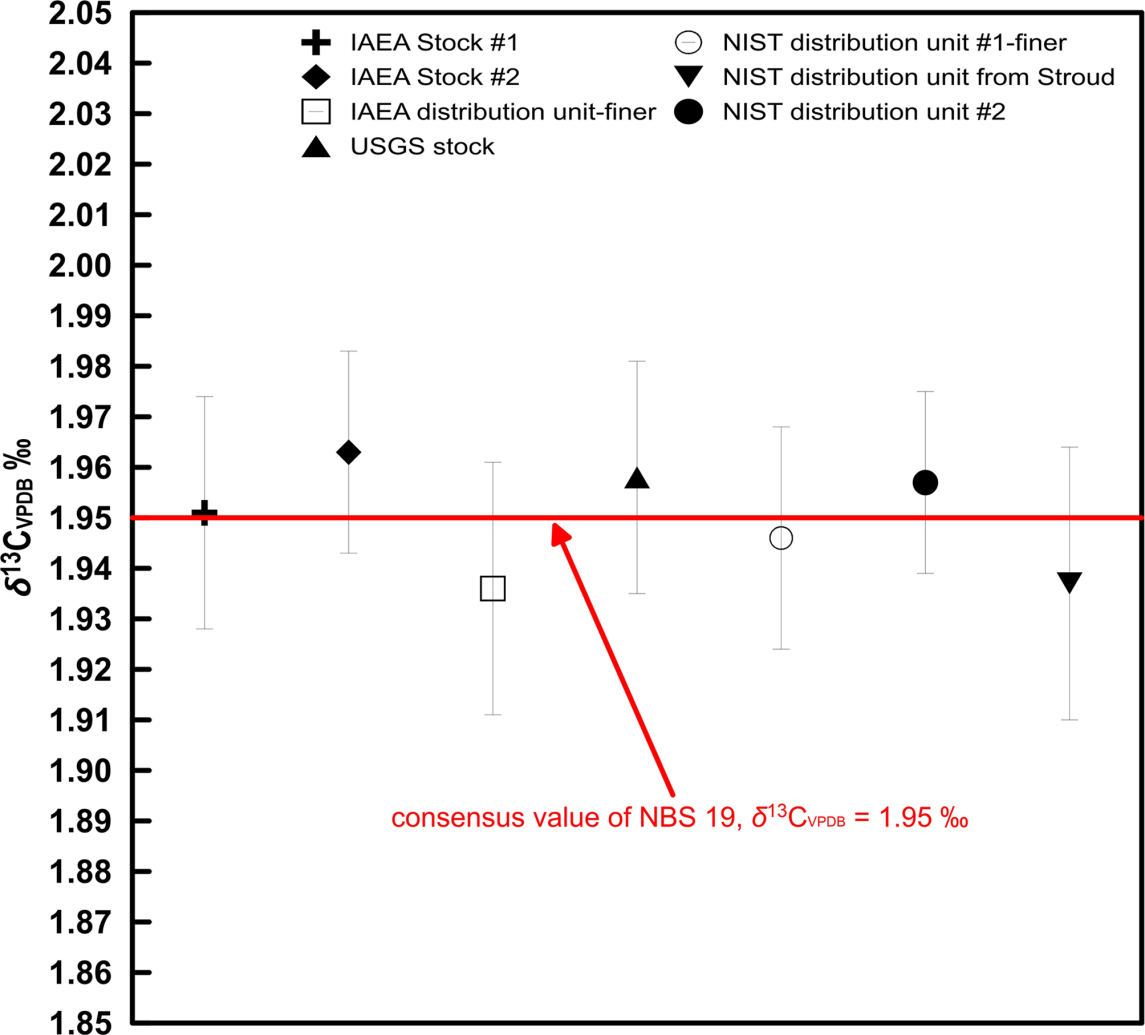
CF‐IRMS *δ* ^13^C_VPDB_ results represent the mean values for each of the seven calcite samples measured across four analytical runs, encompassing a range of NBS 19 limestone stock materials and grain sizes. The uncertainties are the expanded 95% measurement uncertainties (*k* = 2) associated with the δ^13^C_VPDB_ values for these samples (Tables 1 and S6). The laboratory identifiers of these samples are C‐14316 (IAEA Stock #1), C‐14317 (IAEA Stock #2), C‐14318 (IAEA distribution unit‐finer), C‐14319 (USGS stock), C‐14320 (NIST distribution unit #1‐finer), C‐14322 (NIST distribution unit from Stroud), and C‐14321 (NIST distribution unit #2).

**FIGURE 4:**
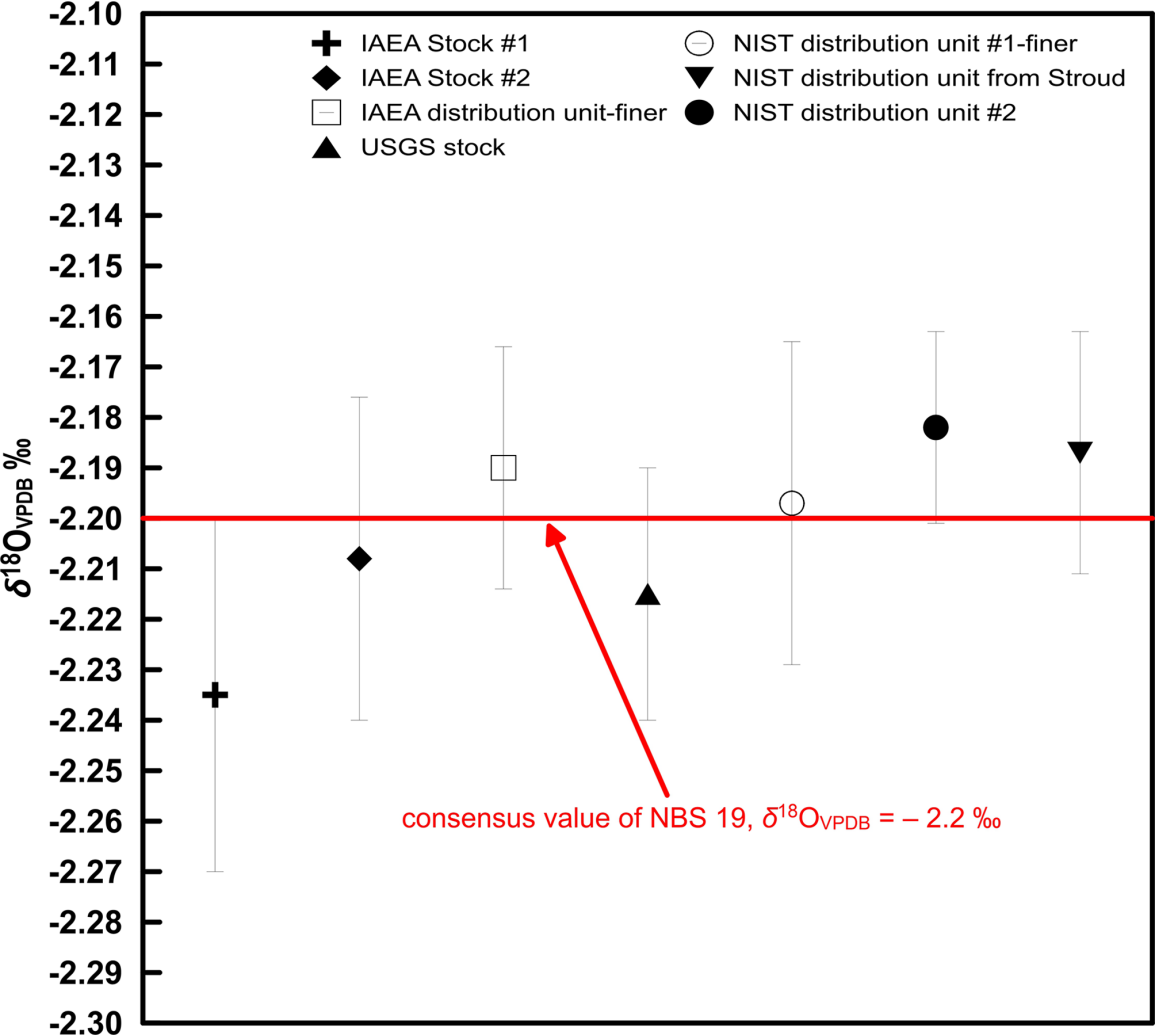
CF‐IRMS *δ* ^18^O_VPDB_ results represent the mean values for each of the seven calcite samples measured across four analytical runs, encompassing a range of NBS 19 limestone stock materials and grain sizes. The uncertainties are the expanded 95% measurement uncertainties (*k* = 2) associated with the *δ* ^18^O_VPDB_ values for these samples (Tables 1 and S6). The laboratory identifiers of these samples are C‐14316 (IAEA Stock #1), C‐14317 (IAEA Stock #2), C‐14318 (IAEA distribution unit‐finer), C‐14319 (USGS stock), C‐14320 (NIST distribution unit #1‐finer), C‐14322 (NIST distribution unit from Stroud), and C‐14321 (NIST distribution unit #2).

A fundamental question in this study is whether stores of NBS 19 limestone at the USGS and the IAEA (Table 1), now quarantined, could have changed in carbon or oxygen isotopic composition owing to differences in storage. An additional question is whether the *δ* ^13^C and *δ* ^18^O values of any of the samples having different grain sizes in Table 1 are distinguishable. To determine whether grain size could affect *δ* ^13^C or *δ* ^18^O of calcite and to investigate whether the same material stored at different locations could lead to variations in *δ* ^13^C and *δ* ^18^O values, four analytical runs were conducted in which seven samples were analyzed repeatedly with masses of 100 or 200 μg. The results are presented in Tables S5 and S6. The expanded 95% measurement uncertainties (*U*_95_; *k* = 2), reflecting laboratory analytical variability of CF‐IRMS measurements of *δ* ^13^C and *δ* ^18^O values, are 0.027‰ and 0.038‰ or better, respectively (Tables 1 and S6). The means and *U*_95_ values of *δ* ^13^C_VPDB_ values of the seven samples (Tables 1 and S6 and Figure 3) encompass the fixed consensus *δ* ^13^C_VPDB_ value of NBS 19 of +1.95‰. Likewise, the means and *U*_95_ values of *δ* ^18^O_VPDB_ values of the same seven samples encompass the fixed consensus *δ* ^18^O_VPDB_ value of NBS 19 of −2.2‰ (Tables 1 and S6 and Figure 4). None of these seven samples in Table 1 and Figures 3 and 4 show a distinguishable *δ* ^13^C and *δ* ^18^O value, confirming that neither storage location nor grain‐size differences have affected the *δ* ^13^C and *δ* ^18^O values of NBS 19.

The standard deviation is a measure of the amount of variation or dispersion in a set of data. It can be applied to multiple samples to compare the variability across the samples. For seven samples analyzed across four runs (130 total individual analyses), the standard deviations of *δ* ^13^C and *δ* ^18^O are 0.047‰ and 0.052‰, respectively (Table S5), for sample masses of 100 and 200 μg. When considering only the seven samples analyzed having masses of 100 μg, the standard deviations are 0.049‰ for *δ* ^13^C values and 0.047‰ for *δ* ^18^O values (Table S5). In comparison, analyses of the same samples having masses of 200 μg yield standard deviations of 0.047‰ for *δ* ^13^C values and 0.054‰ for *δ* ^18^O values. NBS 19 contained between 4 and 8 grains in the 100‐μg samples, whereas the NBS‐19 byproduct samples contained between 13 and 16 grains at the same sample mass. Overall, our results show no evidence of *δ* ^13^C or *δ* ^18^O heterogeneity, even at the 100‐μg scale. These results support the findings of Ishimura et al. [20], who report the following:

Based on the S.D. of the *δ* ^13^C and *δ* ^18^O values determined for CO_2_ gases evolved from the different grains of the same calcite material, we found that NBS 19, IAEA‐CO‐1, and IAEA‐CO‐8 were homogeneous for *δ* ^13^C (less than 0.10‰ S.D.), and that only NBS 19 was homogeneous for *δ* ^18^O (less than 0.14‰ S.D.).

That said, we cannot rule out the possibility of a *δ* ^13^C or *δ* ^18^O difference of 0.01‰ or 0.02‰ between NBS 19 and the secondary isotopic RM NBS‐19 byproduct. Such differences may impact the results of RMs calibrated with NBS‐19 byproduct, for example, IAEA‐603, IAEA‐610, IAEA‐611, and IAEA‐612 [21, 22].

These results indicate that the NBS 19 stored in different locations and in different distribution units and the finer‐than‐80‐mesh TS Marble are indistinguishable with either *δ* ^13^C or *δ* ^18^O CF‐IRMS measurements. The results from 40 aliquot analyses of the same sample from the same vial of NBS 19 limestone (C‐12928 in Tables S1 and S5), which has been utilized at the USGS for decades, demonstrate that the analytical precision of both *δ* ^13^C and *δ* ^18^O measurements of 200‐μg calcite samples is approximately 0.04‰ using the GasBench II‐CF‐IRMS system (Table S5).

### 3.3 Isotopic RMs Calibrated With NBS 19 Limestone

NBS 19 limestone was intentionally created with a large mesh (50‐to‐80; 297 to 177 μm) [7] because it was known that finer grains may change in isotopic composition over time as containers are opened and closed in sometimes moist environments. For example, Hare et al. [23] report a high scatter in the *δ* ^17^O and *δ* ^18^O values of calcite from an almost empty vial of NBS 19 that had been used for 30 years. Therefore, it is encouraged that RMs be calibrated with NBS 19 rather than finer‐than‐80‐mesh TS Marble. Additionally, reaction rates of carbonates may vary with grain size. NBS 19 has been instrumental in calibrating at least 49 isotopic RMs since 2003 (Table 2). RMs not listed in Table 2 may have been calibrated with NBS‐19 byproduct (finer‐than‐80‐mesh TS Marble) secondary isotopic RM, or they may have been calibrated with NBS 19. Confirmation of the grain size of the material is needed for determination. Users can identify the grain size of their calcite by counting the number of grains in a fixed mass. NBS 19 has between 7 and 16 grains in 200‐μg samples, whereas NBS‐19 byproduct has between 25 and 30 grains in 200‐μg samples (Table S4).

**TABLE 2:** Isotopic reference materials known to be calibrated with NBS 19 limestone.

Name	Material	References
IAEA‐CO‐1	Calcite	[16, 17]
IAEA‐CH‐3	Cellulose	[16, 17]
IAEA‐CH‐6	Sucrose	[16, 17]
IAEA‐CH‐7	Polyethylene foil	[16, 17]
IAEA‐CO‐8	Carbonatite	[16, 17]
IAEA‐CO‐9	Barium carbonate	[16, 17]
IAEA‐600	Caffeine	[16, 17]
IAEA‐601, IAEA‐602	Benzoic acid	[16, 17]
NBS 18	Carbonatite	[16, 17]
NBS 22, NBS 22a	Oil, vacuum oil	[16, 17, 24]
USGS24	Graphite	[16, 17]
USGS40, USGS41a	l‐Glutamic acid	[25, 26]
USGS42, USGS43	Human hair	[27]
USGS44	Calcium carbonate	[28]
USGS54, USGS55, USGS56	Wood	[29]
USGS61, USGS62, USGS63	Caffeine	[24]
USGS64, USGS65, USGS66	Glycine	[24]
USGS67, USGS68, USGS69	*n*‐Hexadecane	[24]
USGS70, USGS71, USGS72	Icosanoic acid methyl ester (C20 FAME)	[24]
USGS73, USGS74, USGS75	l‐Valine	[24]
USGS76	Methylheptadecanoate (C17 FAME)	[24]
USGS77	Polyethylene powder	[24]
USGS78	Vacuum oil	[24]
USGS82, USGS83	Honey	[30]
USGS84, USGS85	Olive oil	[30]
USGS86	Peanut oil	[30]
USGS87	Corn oil	[30]
USGS88, USGS89	Collagen	[30]
USGS90, USGS91	Flour	[30]

## 4 Conclusions

The major findings of this investigation include the following:

- Quarantined stocks of USGS‐prepared NBS 19 at the USGS and the IAEA and distribution units from NIST and the IAEA of NBS 19 containing 48‐to‐80‐mesh (297 to 177 μm) calcite are indistinguishable based on CF‐IRMS *δ* ^13^C and *δ* ^18^O measurements.
- Stocks of quarantined NBS 19 residing at the USGS and the IAEA have the same grain size of 48‐to‐80 mesh—originally prepared by Friedman et al. [7]. Although most or all of the calcite labeled “NBS 19” and distributed by NIST and the IAEA in the 1980s and 1990s has a grain size of 48‐to‐80 mesh (297 to 177 μm), both NIST and the IAEA subsequently distributed finer‐than‐80‐mesh (≤ 177 μm) TS Marble, termed NBS‐19 byproduct, until 2011 (Figure S8).
- NBS 19, which has a grain size of 48‐to‐80 mesh by definition, and finer‐than‐80‐mesh TS Marble (NBS‐19 byproduct) are isotopically indistinguishable based on CF‐IRMS measurements of (1) quarantined NBS 19 from stocks at the USGS and the IAEA, (2) NBS 19 from NIST distribution units, (3) a finer‐than‐80‐mesh (≤ 177 μm) TS Marble in a NIST distribution unit, and (4) a finer‐than‐80‐mesh (≤ 177 μm) TS Marble in an IAEA distribution unit. Expanded 95% measurement uncertainties (*k* = 2) for these *δ* ^13^C and *δ* ^18^O determinations are 0.027‰ and 0.038‰ or better, and all mean values (with expanded uncertainties) encompass consensus *δ* ^13^C and *δ* ^18^O values of NBS 19.

Our study reaffirms the stability and reliability of NBS 19 limestone, demonstrated by isotopic analysis of samples obtained from various storage locations and distribution units. The consistent results for *δ* ^13^C and *δ* ^18^O measurements with standard deviations comparable to those of a single isotopically homogeneous calcite specimen underscore the robustness of NBS 19 limestone, regardless of variations in storage conditions. These findings validate the ongoing utility of using NBS 19 as a primary isotopic RM for calibrating new isotopic RMs for decades, thereby reducing traceability chains and reducing calibration uncertainty. Although *δ* ^13^C and *δ* ^18^O measurements of NBS 19 and the finer‐than‐80‐mesh TS Marble (NBS‐19 byproduct) were not distinguishable with current techniques, new technological advances may make them distinguishable. Therefore, future calibrations of new isotopic RMs can be performed with 48‐to‐80‐mesh NBS 19 limestone to follow guidelines in Camin et al. [10, 11]. In addition, preparers can clearly state that finer‐than‐80‐mesh TS Marble secondary isotopic RM was used for calibrations when applicable.

## Author Contributions

**Haiping Qi:** conceptualization, investigation, writing – original draft, validation, writing – review and editing, methodology, formal analysis, data curation, visualization. **Tyler B. Coplen:** conceptualization, investigation, writing – original draft, validation, writing – review and editing, formal analysis, project administration, data curation, supervision. **Lauren T. Reid:** writing – review and editing, data curation, validation. **Stanley J. Mroczkowski:** investigation, validation, writing – review and editing, formal analysis, data curation.

## Data Availability

Data for this study are available in reference [9].
